# Phase 1 randomized trial of a plant-derived virus-like particle vaccine for COVID-19

**DOI:** 10.1038/s41591-021-01370-1

**Published:** 2021-05-18

**Authors:** Brian J. Ward, Philipe Gobeil, Annie Séguin, Judith Atkins, Iohann Boulay, Pierre-Yves Charbonneau, Manon Couture, Marc-André D’Aoust, Jiwanjeet Dhaliwall, Carolyn Finkle, Karen Hager, Asif Mahmood, Alexander Makarkov, Matthew P. Cheng, Stéphane Pillet, Patricia Schimke, Sylvie St-Martin, Sonia Trépanier, Nathalie Landry

**Affiliations:** 1 Medicago Inc., Quebec City, Quebec Canada; 2 Research Institute of the McGill University Health Centre, Montreal, Quebec Canada

**Keywords:** Drug development, Translational research

## Abstract

Several severe acute respiratory syndrome coronavirus 2 (SARS-CoV-2) vaccines are being deployed, but the global need greatly exceeds the supply, and different formulations might be required for specific populations. Here we report Day 42 interim safety and immunogenicity data from an observer-blinded, dose escalation, randomized controlled study of a virus-like particle vaccine candidate produced in plants that displays the SARS-CoV-2 spike glycoprotein (CoVLP: NCT04450004). The co-primary outcomes were the short-term tolerability/safety and immunogenicity of CoVLP formulations assessed by neutralizing antibody (NAb) and cellular responses. Secondary outcomes in this ongoing study include safety and immunogenicity assessments up to 12 months after vaccination. Adults (18–55 years, *n* = 180) were randomized at two sites in Quebec, Canada, to receive two intramuscular doses of CoVLP (3.75 μg, 7.5 μg, and 15 μg) 21 d apart, alone or adjuvanted with AS03 or CpG1018. All formulations were well tolerated, and adverse events after vaccination were generally mild to moderate, transient and highest in the adjuvanted groups. There was no CoVLP dose effect on serum NAbs, but titers increased significantly with both adjuvants. After the second dose, NAbs in the CoVLP + AS03 groups were more than tenfold higher than titers in Coronavirus 2019 convalescent sera. Both spike protein-specific interferon-γ and interleukin-4 cellular responses were also induced. This pre-specified interim analysis supports further evaluation of the CoVLP vaccine candidate.

## Abstract (web summary)

Safety and immunogenicity results in humans of a two-dose SARS-CoV-2 vaccine made from plants support further assessment of potential efficacy.

## Main

Since its emergence in late 2019 (ref. ^1^) and the subsequent declaration of a pandemic by the World Health Organization on 11 March 2020, SARS-CoV-2 has caused more than 130 million cases of Coronavirus Disease 2019 (COVID-19) globally, with more than 2.8 million deaths^2^. Although considerable progress has been made in caring for patients with COVID-19 (refs. ^3,4^), current treatment options remain relatively limited. From the start of the pandemic, there has been a massive global effort to develop vaccines. This effort was primed, to some extent, by previous experience with other highly pathogenic human coronaviruses—SARS-CoV and Middle East respiratory syndrome coronavirus^5^—and the far-sighted efforts of the Coalition for Epidemic Preparedness Innovations to develop vaccines for a ‘short list’ of pathogens with pandemic potential^6^. At the time of this writing, more than 182 vaccine candidates have been announced; more than 81 vaccine candidates are being tested in as many as 230 clinical trials; and 12 vaccines have received authorization for some level of use in at least one jurisdiction^7^. These vaccines and vaccine candidates are remarkable not only in their numbers but also in their diversity, including both traditional (for example, inactivated virion, live attenuated and protein + adjuvant) and novel (for example, mRNA, DNA and replicating and non-replicating viral vectors) platforms^7^.

Adding to the complexity of this situation is the fact that no correlate of protection has been defined for any highly pathogenic coronavirus^8,9^ and the concern that such correlates might differ among vaccines^10^. Nonetheless, a protective role for both humoral and cell-mediated immunity against coronaviruses has been suggested^11,12^. Antibody responses against the spike (S) protein have demonstrated potential to protect from infection in non-human primates^13,14^, and convalescent plasma with high titers of anti-S antibody appear to have therapeutic benefit in selected patients^15,16^. Indeed, there is a growing consensus that NAbs might be a good surrogate for protection^9,14^. Although almost all individuals who contract COVID-19 have detectable antibody responses that last at least 6 months^17,18^, humoral responses in those who are asymptomatic or have mild symptoms can be relatively weak and short-lived, disappearing within months of infection^19–21^, as was observed during the SARS-CoV-1 outbreak of 2002–2003 (ref. ^22^). An effective T cell response might not only be important for recovery from COVID-19 but might also be important for long-term immunity^12,14,23^. Such T cell responses were shown to persist for up to 11 years after SARS-CoV-1 infection^24^, and T cells can provide substantial protection in animal models of highly pathogenic coronavirus infection^25^.

We report here the results of a phase 1 study initiated in July 2020 to evaluate the safety, tolerability and immunogenicity of two doses, 21 d apart, of 3.75 µg, 7.5 µg or 15 µg of a plant-produced virus-like particle (VLP) vaccine candidate (hereafter called CoVLP) for COVID-19. VLP vaccines have been highly successful for several viral pathogens (for example, hepatitis B virus and human papilloma virus), possibly owing to their ability to effectively deliver the targeted antigens to the immune system and to stimulate both humoral and cellular (that is, CD4^+^ T cell) responses^26^. Medicago’s plant-based production platform uses transient transfection of *Nicotiana benthamiana*, a common Australian plant, and a disarmed *Agrobacterium tumefaciens* vector to deliver the foreign episomal DNA to the plant cell nucleus (see Methods for further details)^27,28^. The CoVLP vaccine can be stored at 2–8 °C, and its stability was closely monitored throughout the trial.

This platform has also been used to produce hemagglutinin-bearing VLP vaccines for avian (monovalent) and seasonal (quadrivalent) influenza that induce balanced humoral and T cell responses^29–32^. The CoVLP vaccine was administered alone or with AS03 or CpG1018 adjuvants in healthy adults 18–55 years of age. To our knowledge, this is the first report of a clinical trial of a candidate, plant-derived vaccine for COVID-19, and it follows in the footsteps of a small number of other successful uses of plant-based platforms to produce biotherapeutics (for example, taliglucerase alpha for Gaucher’s disease and monoclonal antibodies for Ebola)^33,34^.

## Results

### Demographic and baseline clinical characteristics

**Fig. 1:**
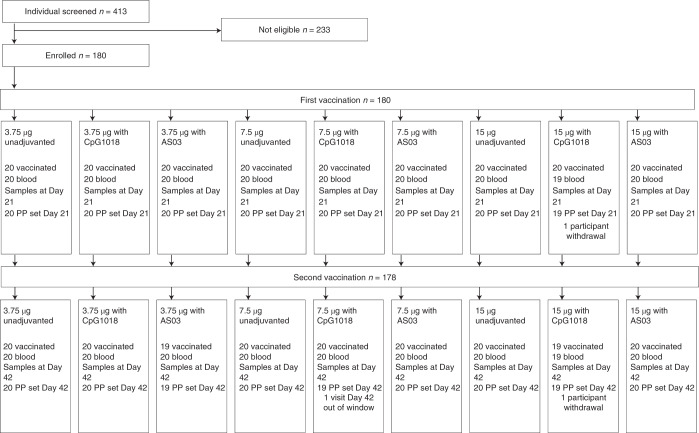
Trial profile—participant disposition. Enrollment and follow-up of study participants vaccinated with 3.75 µg, 7.5 µg or 15 µg CoVLP with or without AS03 or CpG1018 adjuvant after the first and second dose administration. One participant in the 3.75 µg + AS03 treatment group did not receive the second vaccination as per protocol owing to a grade 3 AE (fatigue) after the first dose but agreed to have blood collection for immunogenicity. One participant in the 15 µg + CpG1018 treatment group withdrew consent before the second vaccination and consequently did not have blood collection at Day 21 and Day 42. Both participants were excluded from the per-protocol set. For more details of participant disposition, see Table 1. PP, per-protocol.

Participants were screened for SARS-CoV-2 antibodies at two Canadian phase 1 trial sites located in Montreal (*n* = 47) and Quebec City (*n* = 133) using a commercial enzyme-linked immunosorbent assay (ELISA) that targets the nucleocapsid (N) protein (Elecsys, Roche Diagnostics), and only seronegative individuals were included. Participant demographics are presented in Table 1, and participant disposition to Day 42 is presented in Fig. 1 and Supplementary Table 4. None had participated in a prior study conducted by Medicago. Among the 180 participants enrolled, there were slightly more women (56.7%) than men (43.3%), but the female:male ratio was roughly the same for each CoVLP dose level tested (3.75 μg, 7.5 μg and 15 μg) and in each of the three formulation groups (unadjuvanted CoVLP, CoVLP + AS03 and CoVLP + CpG1018); hence, 20 participants were randomized to each of nine groups. Participants were mostly White (96%) with 2% each of Black or African American and Asian participants. The average age was 34.3 years. Of the 180 participants who received the first dose of vaccine, 178 also received the second dose.

**Table 1:** Summary of demographics and baseline characteristics (NCT04450004)

	CoVLP 3.75 µg			CoVLP 7.5 µg			CoVLP 15 µg			All
	Unadjuvanted	Adjuvanted with CpG 1018	Adjuvanted with AS03	Unadjuvanted	Adjuvanted with CpG 1018	Adjuvanted with AS03	Unadjuvanted	Adjuvanted with CpG 1018	Adjuvanted with AS03	
Participants	20	20	20	20	20	20	20	20	20	180
Sex, *n* (%)										
Male	9 (45.0)	10 (50.0)	5 (25.0)	10 (50.0)	8 (40.0)	8 (40.0)	7 (35.0)	10 (50.0)	11 (55.0)	78 (43.3)
Female	11 (55.0)	10 (50.0)	15 (75.0)	10 (50.0)	12 (60.0)	12 (60.0)	13 (65.0)	10 (50.0)	9 (45.0)	102 (56.7)
Race, *n* (%)										
White	18 (90.0)	20 (100.0)	20 (100.0)	20 (100.0)	18 (90.0)	19 (95.0)	20 (100.0)	18 (90.0)	19 (95.0)	172 (95.6)
Black or African American	1 (5.0)	0 (0.0)	0 (0.0)	0 (0.0)	1 (5.0)	0 (0.0)	0 (0.0)	1 (5.0)	1 (5.0)	4 (2.2)
Asian	1 (5.0)	0 (0.0)	0 (0.0)	0 (0.0)	1 (5.0)	1 (5.0)	0 (0.0)	1 (5.0)	0 (0.0)	4 (2.2)
Ethnicity, *n* (%)										
Hispanic/Latinx	0 (0.0)	2 (10.0)	0 (0.0)	1 (5.0)	3 (15.0)	1 (5.0)	0 (0.0)	1 (5.0)	0 (0.0)	9 (5.0)
Age at vaccination										
Mean ± s.d.	34.9 ± 8.3	35.3 ± 11.0	34.7 ± 9.1	35.6 ± 8.0	32.4 ± 9.5	37.2 ± 7.8	34.1 ± 9.6	32.0 ± 9.0	32.7 ± 9.1	34.3 ± 9.0
Median (range)	35 (18–49)	36 (18–53)	36 (19–49)	36 (20–50)	31 (19–52)	37 (21–55)	31.5 (22–54)	30 (19–51)	32.5 (18–52)	34 (18–55)

### CoVLP vaccine

The candidate vaccine is described in more detail in Methods. Briefly, the full-length S glycoprotein in a stabilized, pre-fusion configuration is expressed in *N. benthamiana*. Spontaneously assembled VLPs bud off the plant cell surface and accumulate in the virtual space between the plasma membrane and the cell wall. Electron microscopy revealed CoVLPs to be nanoparticles similar in size (80–120 nM) and shape to the SARS-CoV-2 virus, comprised of a lipid envelope derived from the plant cell plasma membrane in which S protein trimers appear to be anchored at high density based on electron micrograph images (Supplementary Fig. 1).

### Safety

**Fig. 2:**
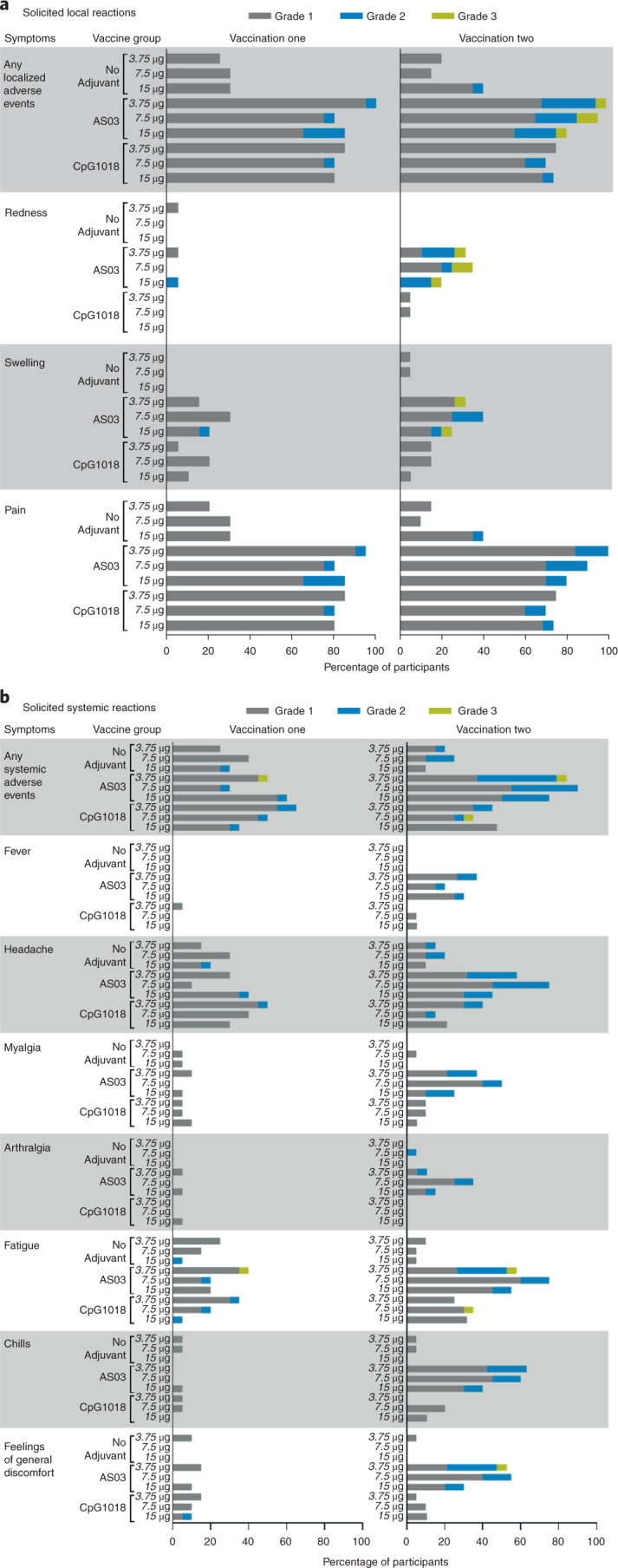
Solicited local and systemic AEs 7 d after the first or second vaccine dose. Participants were monitored for solicited local (**a**) and systemic (**b**) AEs from the time of vaccination through 7 d after vaccine administration. There was no grade 4 (potentially life-threatening) event. Participants who reported no AEs make up the remainder of the 100% calculation (data not shown). If any of the solicited AEs persisted beyond Day 7 after each vaccination (when applicable), it was recorded as an unsolicited AE. Fever was defined as oral temperature ≥38.0 °C.

Local and systemic solicited adverse events (AEs) and other safety data were collected during a 15-min observation period after each intramuscular vaccination, during two telephone contacts on Day 1 and Day 8 and during a site visit on Day 3. Reactogenicity for all formulations is shown in Fig. 2a,b. AEs were mostly mild to moderate (grades 1 and 2) with only one grade 3 event of fatigue that started the evening after vaccination and resolved the same day. Both adjuvants increased the frequency of reported AEs. The frequency and severity of AEs were similar after the first and second doses in the unadjuvanted CoVLP and CoVLP + CpG1018 groups but tended to increase after the second dose in participants who received AS03-adjuvanted formulations. Details of solicited/unsolicited AEs by treatment group are provided in Supplementary Tables 4–9. There was no consistent effect of CoVLP dose level on safety outcomes. After the first dose, 74.3% of participants reported more than one solicited AE; 66.5% reported a local reaction; and 39.7% reported more than one systemic event. Pain at the injection site was the most common local reaction (66.5%), and headache and fatigue were reported by 25.7% and 20.7%, respectively. The incidences of headache and fatigue were generally higher in the adjuvanted treatment groups. After the second dose, 68.5% of participants reported more than one solicited AE; 62.9% reported a local reaction; and 47.8% reported more than one systemic event. Pain at the injection site was, again, the most reported local reaction (61.2%), and headache and fatigue were reported by 33.1% and 33.1%, respectively. Again, most symptoms were mild, but there were more moderate AEs after the second dose. Nine grade 3 solicited AEs (fatigue, redness at injection site, swelling at injection site and feeling of general discomfort or uneasiness) were reported in six participants after the second dose. All but one of the grade 3 reactions were reported by participants who received AS03-adjuvanted formulations. One grade 3 reaction occurred after the second dose in the CoVLP 7.5 μg + CpG1018 group. All grade 3 AEs resolved in 1–4 d. No grade 4 solicited reactions were reported. Five unsolicited grade 4 cases and one grade 3 case of increased serum creatine phosphokinase (CPK) were reported (two in CoVLP 15 μg + AS03 and two in CoVLP 7.5 μg + CpG1018 and one each in CoVLP 3.75 μg unadjuvanted and CoVLP 7.5 μg unadjuvanted) (see footnotes in Supplementary Tables 5–7). All of these events occurred in participants with other obvious reasons for the CPK elevations (for example, strenuous or unaccustomed physical activity immediately before vaccination) and were assessed by the investigator as unrelated to the study vaccines. All AEs were monitored closely by the Independent Data Monitoring Committee (IDMC) and resolved rapidly. No other clinically significant laboratory abnormalities, serious adverse events (SAEs) or adverse events of special interest (AESIs) considered to be related to the vaccine were reported with any formulation after the first or second dose. No pregnancies have been reported to date. A total of 12 IDMC scheduled meetings have occurred during this study to date, with no safety signal identified.

### Immunogenicity: antibody response

**Fig. 3:**
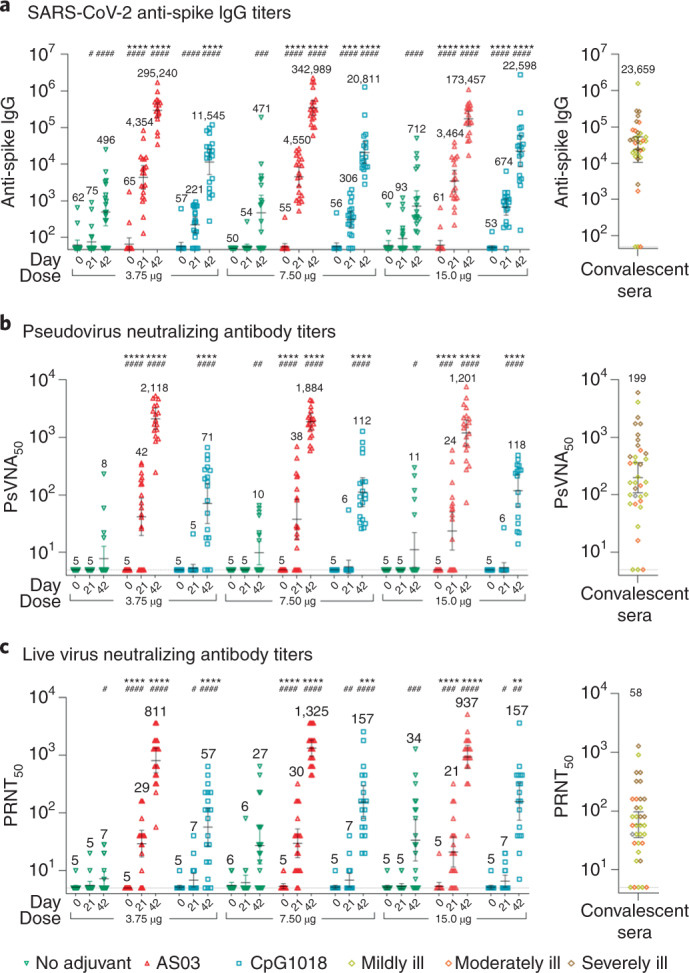
Humoral response to CoVLP alone or with adjuvants. Serum antibodies of participants vaccinated with 3.75 µg, 7.5 µg or 15 µg CoVLP with or without AS03 or CpG1018 adjuvant were measured to S protein by ELISA (**a**) and in neutralization assays based on a VSV pseudovirus (**b**) or live virus (**c**) and presented here as reciprocal titers. Inverted green triangles are used for unadjuvanted CoVLP groups; upright red triangles are used for the CoVLP + AS03 groups; and blue squares are used for the CoVLP + CpG1018 groups. Convalescent sera or plasma were collected at least 14 d after a positive diagnosis of COVID-19 (RT–PCR) from individuals whose illness was classified as mild, moderate or severe/critical (*n* = 35). These samples were analyzed in the anti-S ELISA and both neutralization assays; results (right panels). Horizontal bars and numbers in the figure indicate geometric means. Error bars indicate 95% CIs. Significant differences among Days 0 and 21 or Days 0 and 42 for each formulation are indicated by a hashtag (#*P* < 0.05, ##*P* < 0.01, ###*P* < 0.001, ####*P* < 0.0001; paired two-sided *t*-test of log-transformed values, GraphPad Prism v8.1.1). Significant differences between unadjuvanted and adjuvanted regimens for Days 21 and 42 are indicated by an asterisk (*P < 0.05, **P < 0.01, ***P < 0.001, ****P < 0.0001; two-way ANOVA of log-transformed values, GraphPad Prism v8.1.1). The PsVNA_50_ is reciprocal of the serum dilution at which a decrease in luminescence ≥50% was observed in the PNA. The PRNT_50_ is the reciprocal serum dilution at which ≥50% of the cells were free from infection in the MNA.

As illustrated in Fig. 3, unadjuvanted CoVLP elicited no detectable antibody response after the first dose, and humoral responses after even the second dose were modest and inconsistent. Although a minor dose effect for the unadjuvanted CoVLP was seen on the anti-S IgG response (ELISA) after the second vaccination, the responses in the two NAb assays remained low and variable even at the highest dose tested (15 μg). Both adjuvants had a significant effect on antibody responses at all CoVLP dose levels. Although there was no significant dose effect in the adjuvanted groups, responses were more consistent in the CoVLP + CpG1018 groups at higher CoVLP doses (that is, trends to a greater response and a larger proportion of participants responding). Although both adjuvants elicited readily detectable IgG titers after the first dose, only the groups that received CoVLP + AS03 formulations mounted significant NAb responses at Day 21 (36/60, 60%) across all dose levels (that is, overall geometric mean titer (GMT) of 33.3 in the pseudovirion neutralization assay (PNA) and 26.4 in the microneutralization assay (MNA): *P* < .0001 versus unadjuvanted formulations in both assays). Both adjuvants induced more robust responses after the second dose, with the large majority of participants at all dose levels mounting a fourfold or greater rise in total IgG (117/118, 99.1%) and in both NAb assays (105/112 (93.8%) in the PNA and 106/116 (91.3%) in the MNA: the mean fold rise in the NAb assays between the first and second dose ranged from 8- to 18-fold in the CpG1018-adjuvanted groups and between 28- and 92-fold in the AS03-adjuvanted groups) (Fig. 3). At all dose levels, the anti-S IgG and NAb titers in both of the neutralization assays used at Day 42 were 6–29× higher in participants who had received AS03-adjuvanted formulations compared to those who had received CpG1018-adjuvanted formulations. For example, after the second immunization at the 3.75-μg dose level, the GMTs in the MNA were 7.2 for CoVLP alone, 56.6 for CoVLP + CpG1018 and 811.3 for CoVLP + AS03 (*P* < .0001 for both adjuvanted formulations versus unadjuvanted). Overall, the levels of NAb induced in the groups that received two doses of CoVLP with an adjuvant were either similar to (CoVLP + CpG1018) or substantially greater than (CoVLP + AS03) those seen in participants recovering from natural COVID-19 infection (16 mild, 8 moderate and 11 severe/critical; see Supplementary Table 3 for details of convalescent samples and Supplementary Tables 11–13 for serologic results in convalescent patients with mild, moderate or severe presentations). After the second dose, 100% of participants who received AS03-adjuvanted formulation seroconverted in the anti-spike IgG ELISA and both neutralization assays, regardless of the CoVLP dose level (Fig. 3).

A small number of participants (12/180, 6.7%) appeared to have detectable anti-S protein antibodies on Day 0 in one or more of the assays used, despite having tested negative in the N protein assay used at screening. Baseline serostatus had no significant effect on the Day 42 serologic responses to vaccination (data not shown). Details of serologic response results by treatment group are presented in Supplementary Tables 11–13. There was a strong correlation between the vesicular stomatitis virus (VSV) PNA and MNA responses: *r* = 0.84 after the first vaccination and *r* = 0.88 after the second vaccination (both *P* < 0.0001; Supplementary Fig. 2). A small proportion of samples (~2%) yielded discrepant results in the two neutralizing assays (below the lower limit of detection in the PNA and low-positive in the MNA), suggesting higher sensitivity of the latter assay.

### Immunogenicity: T cell response

**Fig. 4:**
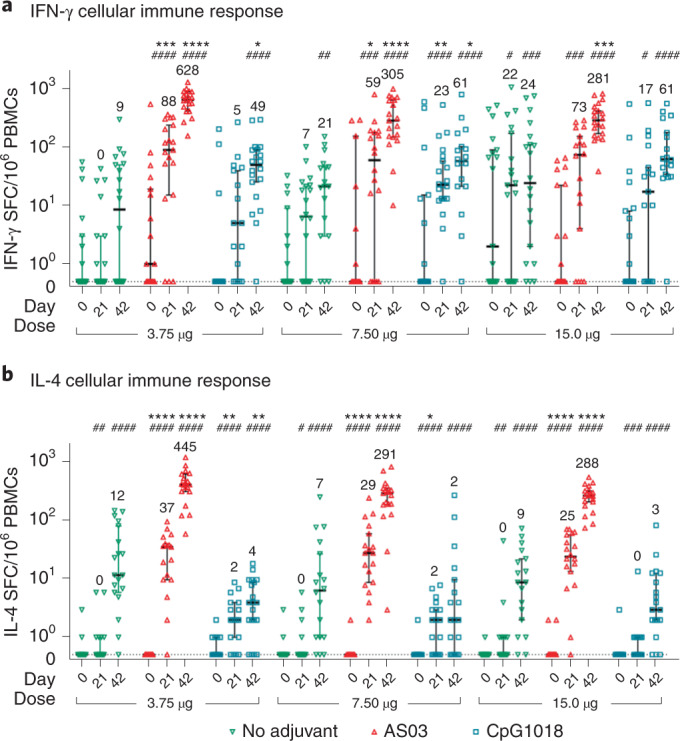
Cellular immune response to CoVLP alone or with adjuvants. Frequencies of S protein-specific production of IFN-γ (**a**) or IL-4 (**b**) at baseline (Day 0) and 21 d after the first (Day 21) or second (Day 42) vaccine dose with 3.75-µg, 7.5-µg or 15-µg doses of CoVLP with or without adjuvants (CpG1018 and AS03). PBMCs were stimulated ex vivo with a peptide pool covering the entire S protein (15-mers with 11-amino acid overlap). Bars and numbers in the figure indicate group medians, and error bars indicate 95% CI. The median pre-vaccination values in both assays across all groups was 0. Significant differences between Day 0 and Day 21 or between Day 0 and Day 42 for each vaccine regimen are indicated by a hashtag (^#^*P* < 0.05, ^##^*P* < 0.01, ^###^*P* < 0.001, ^####^*P* < 0.0001; unpaired two-sided *t*-test; the figure illustrates matched subject data, GraphPad Prism v8.1.1). Significant differences among adjuvanted vaccine and unadjuvanted vaccine regimens at Day 21 and Day 42 are indicated by an asterisk (*P < 0.05, **P < 0.01, ***P < 0.001, ****P < 0.0001; Kruskal–Wallis test, GraphPad Prism v8.1.1). SPC, spot-forming cell.

As illustrated in Fig. 4, the interferon (IFN)-γ and interleukin (IL)-4 responses in peripheral blood mononuclear cells (PBMCs) (enzyme-linked immunospot (ELISpot)) elicited by CoVLP with and without adjuvants were more variable than the antibody responses. In most treatment groups, a minority of participants in each group (mean ± s.d.: 7.2% ± 2.9%) had pre-existing IFN-γ responses to the S protein peptide pool that were, in some cases, substantial (that is, >200 spots), as was previously reported^35,36^. Although low-level ‘background’ IL-4 activity was seen in a small number of participants, these responses were close to the limit of detection of the assay used. Unlike antibody responses, CoVLP alone induced substantial IFN-γ (32/55 with ≥10 spots (58%)) and IL-4 (26/53 with ≥10 spots (49%)) responses after the second doses (*P* values ranging from not significant to *P* < 0.001 for IFN-γ at different dose levels and consistently *P* < 0.0001 for IL-4 at all dose levels). Both adjuvants increased IFN-γ and IL-4 responses above background levels after the first dose that were further increased in both magnitude and consistency (that is, the proportion of participants responding) by the second dose. Once again, the IFN-γ and IL-4 responses to the CoVLP + AS03 formulations at all dose levels were 10 –50× higher than those seen in the equivalent unadjuvanted group. CoVLP + CpG1018 responses were approximately 5× higher than the unadjuvanted groups for IFN-γ and similar or reduced for IL-4. For example, at the 3.75-µg dose level, median IFN-γ and IL-4 responses at Day 42 were 628 and 445, respectively, in the CoVLP + AS03 group and 49 and 4, respectively, in the CoVLP + CpG1018 group (IFN-γ response versus unadjuvanted *P* < 0.0001 for CoVLP + AS03 and *P* < 0.05 for CoVLP+CpG1018; IL-4 response versus unadjuvanted *P* < 0.001 for CoVLP+AS03 and *P* < 0.01 for CoVLP + CpG1018). The presence of a detectable IFN-γ response to the S protein peptide pool on Day 0 had no significant effect on the Day 21 or Day 42 cellular responses in any group. Details of cellular response results by treatment group are provided in Supplementary Tables 14 and 15.

## Discussion

This study was designed to select the CoVLP formulation (that is, dose level with or without adjuvant) and the number of doses needed to generate a consistent immune response in healthy adults with an acceptable safety profile. Although participants in this trial will be followed for 12 months after the second immunization, the primary study outcomes were focused on tolerability/safety and immunogenicity of the vaccine formulations up to 21 d after each dose. The unadjuvanted CoVLP formulation had the lowest reactogenicity, but the immune responses measured (that is, anti-S IgG, MNA, PNA, IFN-γ and IL-4 ELISpots) were generally modest. Although there was little apparent dose effect with unadjuvanted CoVLP for either antibody or cellular responses, it is possible that higher doses would have induced stronger responses. The two adjuvants incorporated into this study were included precisely for their dose-sparing potential, and most of the measured immune outcomes in participants who received the lowest CoVLP dose with either adjuvant were many-fold greater than the responses seen in even the highest unadjuvanted CoVLP group.

The inclusion of CpG1018 and AS03 permitted several questions to be answered simultaneously. Based on their performance characteristics^37–40^, both adjuvants were expected not only to permit dose-sparing but also to increase the overall magnitude of the immune response. By targeting innate toll-like receptor 9, a T helper (Th1)-type response was anticipated with CpG1018 (ref. ^41^), whereas the combination of squalene and tocopherol (vitamin E) in AS03 was expected to simulate a more balanced Th1/Th2-type response^42–44^. Administered with CoVLP, AS03 proved to be more effective than CpG1018 in both dose-sparing and enhancing responses. For example, as measured by the MNA response, 15/20 (75%) of the participants who received even the lowest dose of CoVLP + AS03 (3.75 µg) seroconverted after the first dose, but only 2/20 (10%) in the equivalent-dose CpG1018-adjuvanted group seroconverted. Furthermore, the anti-S IgG and NAb responses were consistently higher at all CoVLP dose levels in the AS03 groups compared to the CpG1018 groups. Differences between unadjuvanted and adjuvanted formulations and between the two adjuvants were less pronounced in the IFN-γ ELISpot than for antibody responses, suggesting that CoVLP alone can stimulate a cellular response. The ability of CoVLP by itself to induce a degree of cellular immunity is consistent with extensive studies of T cell responses to plant-derived influenza vaccine candidates^29,31,32^. Nonetheless, the IFN-γ ELISpot responses to adjuvanted formulations were consistently higher at most CoVLP dose levels compared to CoVLP alone, particularly after the second dose. Again, IFN-γ responses were strongest in the AS03-adjuvanted groups, particularly at lower CoVLP dose levels. Although such IFN-γ responses could theoretically be mediated by other cells (for example, natural killer or CD8 T cells)^43^, our previous observations with plant-derived influenza vaccine candidates^29–32,45–47^ and recent experience with an AS03-adjuvanted S protein vaccine in non-human primates^48^ suggest that this production is most likely attributable to CD4^+^ T cells. The IL-4 ELISpot responses were also consistently higher in the AS03-adjuvanted groups than the unadjuvanted and CpG1018-adjuvanted groups, rising to near equivalence with the IFN-γ response with the second dose. These early immune outcome data suggest that, at the dose levels tested, CoVLP alone elicited a modest Th1-biased response and that this pattern of response was reinforced by CpG1018. In contrast, when CoVLP was adjuvanted with AS03, the response was faster and more balanced with evidence of both Th1- and Th2-type activation. These differential effects of the adjuvants are generally consistent with a recent study that compared responses to a recombinant S protein administered with several adjuvants in non-human primates, including AS03 and CpG1018 + alum^48^, as well our own observations in Indian rhesus macaques (S.P. et al., unpublished data). Although Th2-deviated responses have been implicated in vaccine-associated enhanced disease (VAED) with some antigens (for example, the initial formalin-inactivated respiratory syncytial virus vaccine)^49^ and are a theoretical risk for vaccines targeting pathogenic coronaviruses^50^, the engagement of IL-4-producing T helper follicular cells is likely to be a critical factor in germinal center formation, B cell maturation and induction of strong and durable antibody responses during viral infection^51^. Furthermore, there has been no evidence to date of VAED in either the animal models of challenge after vaccination or the rapidly growing number of human trials^52^. This might be due, at least in part, to the fact that, unlike some other highly pathogenic coronaviruses, SARS-CoV-2 does not productively infect human macrophages^53^.

It is noteworthy that a substantial proportion of the participants in this study appeared to have pre-existing IFN-γ responses to the S protein peptide pool used for PBMC re-stimulation. Such cross-reactive T cell memory, possibly due to previous exposure to common human coronaviruses, has been seen in 40–60% of adults and might provide some protection against highly pathogenic strains^35,36^. Previous exposure to circulating coronaviruses might also explain the small number of participants in this study (6.7%) who were ‘seronegative’ at screening based on a commercial N protein-based ELISA but who were ‘seropositive’ at Day 0 in our assays that targeted the S protein. The presence of possible cross-reactive antibodies and/or responding T cells at Day 0 did not have a significant effect on the responses to vaccination.

The safety profile of CoVLP alone was relatively benign at all dose levels, but, as expected, both the frequency and the intensity of local and systemic AEs were increased with either adjuvant. Compared to the first dose, the frequency of grade 2 or grade 3 AEs also increased after the second dose in all groups that received adjuvanted formulations. All AEs reported, regardless of dose level, adjuvant status or initial intensity, were transient and resolved rapidly. Although one participant did not receive a second dose of vaccine as per protocol owing to a transient grade 3 AE (fatigue) after the first dose, and a second participant withdrew for personal reasons, no participant withdrew from the study as a result of an AE/SAE. Overall, the reactogenicity of the adjuvanted CoVLP formulations was similar to that reported for several other candidate SARS-CoV-2 vaccines in their early-phase clinical studies^7^. Although hypersensitivity to plant material is a theoretical risk with any plant-derived product, no participant in the current study had an allergic-type reaction to the investigational product, consistent with Medicago’s safety database of more than 14,000 individuals who have received one or two doses of plant-derived influenza vaccine candidates^54^.

Like any early-phase clinical trial, this study had several limitations beyond the obvious concern regarding small group size when testing multiple dose levels and formulations (*n* = 20 per group). For comparisons among formulations, however, this risk was mitigated, to some extent, by the fact that results across the three CoVLP dose levels were quite consistent (*n* = 60 for CoVLP alone or with each adjuvant). Other obvious concerns that apply to all early-phase trials of COVID-19 vaccines are the lack of reference reagents or standardized assays to permit comparisons across studies, the absence of well-defined correlates of immunity and no simple definition of VAED^55^. Regarding reagents, assays and correlates, it is reassuring that the adjuvanted CoVLP formulations elicited NAb responses that were at least as high as those seen in convalescent serum/plasma, including the characteristically higher responses seen in hospitalized patients, as well as strong T cell responses. If one accepts the results of convalescent serum/plasma in different assays as a tentative ‘yardstick’ with which to compare results among studies^56^, it is promising that the NAb responses induced by adjuvanted CoVLP formulations were at least as high as those seen in early-phase reports of vaccines that have released efficacy results^7^. Like all other early-phase trials, our study assessed only the short-term (Day 42) response to vaccination, and the durability of these responses will only become apparent as particpants are followed for longer periods of time. Based on the robust antibody and balanced cellular responses to adjuvanted CoVLP formulations and our previous experience with plant-derived VLP vaccines targeting influenza virus, we are hopeful that CoVLP-induced responses will be long-lived^29–32,57^. The data presented herein reflect the primary study outcomes, but the collection of safety data (that is, SAEs, AESIs and potential immune-mediated diseases (pIMDs)) and immunogenicity data (S-binding IgG, MNA and PNA and ELISpots for IFN-γ and IL-4) will continue for 12 months after administration of the second dose for the secondary outcomes. Finally, the large majority of participants in this study (82%) were recruited at a phase 1 study site located in Quebec City that has low diversity (<5% self-reported ethnic minorities). The ongoing phase 2/3 efficacy trial of CoVLP + AS03 is a global study being conducted in up to 11 countries in North, Central and South America and Europe to ensure a greater level of diversity.

In conclusion, this first trial of CoVLP alone or adjuvanted with either CpG1018 or AS03 suggests that this plant-derived VLP vaccine candidate was well tolerated and immunogenic. Its immunogenicity, particularly at low doses, was markedly enhanced by the presence of an adjuvant, achieving NAb levels at least similar to those seen in individuals recovering from COVID-19. These data are consistent with our unpublished observations in a large study in Indian macaques in which two doses of CoVLP (15 μg) with AS03 induced strong NAb and CD4^+^ T cell responses and reduced viral loads in respiratory tissues with no evidence of vaccine-enhanced disease upon challenge (S.P. et al., unpublished data). Given the growing interest in the possible advantages of heterologous prime-boost vaccination strategies^57^, the recent emergence of SARS-CoV-2 variants that are relatively resistant to neutralization^58^ and the high NAb titers elicited by CoVLP + AS03 (~10- to 14-fold higher than those seen in convalescent patients), the further development of this formulation is strongly supported. Based on the available data and the advantages of dose sparing in a pandemic, a two-dose schedule of CoVLP at 3.75 μg per dose adjuvanted with AS03 has been carried forward into ongoing phase 2/3 studies in Canada and the United States, with planned expansion to additional countries in Latin America and Europe.

## Methods

### CoVLP vaccine and adjuvants

The full-length S glycoprotein of SARS-CoV-2, strain hCoV-19/USA/CA2/2020, corresponding in sequence to nucleotides 21,563–25,384 from EPI_ISL_406036 in the GISAID database (https://www.gisaid.org/), was expressed in *N. benthamiana* plants using *A. tumifaciens* transfection, and the downstream purification processes were very similar to those previously described to produce VLPs bearing influenza hemagglutinin proteins^27^. In this system, S protein expression is not plasmid driven per se. Rather, the *Agrobacterium* vector cuts a defined segment of the plasmid and transfers it to the nucleus of the plant cells. This segment remains episomal for some time before being degraded (hence, transient expression) and drives the expression of S protein. For the CoVLP vaccine candidate, the S protein was modified with R667G, R668S and R670S substitutions at the S1/S2 cleavage site to increase stability and K971P and V972P substitutions to stabilize the protein in pre-fusion conformation^59^. The signal peptide was replaced with the protein disulfide isomerase from alfalfa, and the transmembrane (TM) domain and cytoplasmic tail (CT) of S protein were replaced with TM/CT from influenza H5 A/Indonesia/5/2005 to increase VLP assembly and budding^60,61^. Expression of the S protein was driven using the double 35S promoter and proprietary 5′ and 3′ untranslated regions developed to maximize mRNA stability and protein translation. The TBSV P19 suppressor of gene silencing, used under license from Plant Bioscience Limited, is co-expressed to maximize the transient expression of S protein. The self-assembled VLPs bearing S protein trimers were isolated from the plant matrix and subsequently purified using a process similar to that described for the influenza vaccine candidates^29,60,61^. Briefly, *N. benthamiana* plants were grown in a controlled greenhouse environment for approximately 5 weeks before being exposed to the *A. tumefaciens* transfer vector by vacuum infiltration. After infiltration, plants were placed in a growth chamber under optimal conditions for CoVLP production for up to 6 d. Aerial parts of the plants were then harvested, and the VLPs were released using a proprietary extraction method. The bulk drug substance containing concentrated CoVLPs was then purified using a series of standard industrial filtration and chromatography unit operations steps. The AS03 adjuvant, an oil-in-water emulsion containing DL-α-tocopherol (11.69 mg per dose) and squalene (10.86 mg per dose), was supplied by GlaxoSmithKline. The CpG1018 adjuvant, composed of cytosine phosphoguanine (CpG) motifs (3 mg per dose), was supplied by Dynavax.

### Vaccine preparation and injection

For this study, the CoVLP vaccine was available in single-dose vials (0.30 ml) at concentrations of 15 μg ml^−1^, 30 μg ml^−1^ and 60 μgml^−1^ and was stored at 2–8 °C until shortly before use. The AS03 adjuvant was supplied in multi-dose vials (ten doses per vial) containing DL-α-tocopherol (53.76 mg ml^−1^) and squalene (43.44 mg ml^−1^); however, each AS03 vial was used only once—that is, to prepare one dose of the adjuvanted formulation— and then was discarded. The AS03 vials were stored at 2–8 °C until shortly before use. The CpG1018 adjuvant was supplied as single-dose vials containing CpG motifs at 6 mg ml^−1^ and was stored at 2–8 °C until used. Immediately before use, CoVLP with or without the appropriate adjuvant was brought to room temperature. For the AS03 + CoVLP group, 0.30 ml of CoVLP and 0.30 ml of AS03 were gently mixed 1:1 volume:volume in the CoVLP vaccine vial, and a dose for injection was withdrawn (final dose: 3.75 μg, 7.5 μg or 15 μg of CoVLP + 0.25 ml of AS03 per dose in a volume of 0.50 ml). For the CoVLP + CpG1018 group, 0.30 ml of the CoVLP vaccine was gently mixed with 0.60 ml of the CpG1018 adjuvant, and doses for injection were withdrawn (final dose: 3.75 μg, 7.5 μg or 15 μg CoVLP + 3 mg of CpG1018 per dose in a volume of 0.75 ml). For the CoVLP group, doses for injection were withdrawn directly from the single-dose vial (final dose: 3.75 μg, 7.5 μg or 15 μg of CoVLP in a volume of 0.25 ml). All injections were administered intramuscularly using a 23-gauge needle in the deltoid. The first and second doses were administered contralaterally.

### Study design

This phase 1 randomized controlled trial was conducted at two sites in Quebec City (Syneos Health Clinique Inc.) and Montreal (Syneos Health Clinique Inc.) (see the full protocol in the Supplementary Information for details). The study was approved by a central research ethics review board as well as the Health Products and Food Branch of Health Canada and was carried out in accordance with the Declaration of Helsinki and the principles of Good Clinical Practices. Participants were recruited from existing databases of volunteers, and written informed consent was obtained from all study participants before any study procedure. Participants were offered modest compensation for their participation in this study (that is, time off work and displacement costs). At screening, health status was assessed by medical history, physical examination and clinical laboratory findings, including detection of anti-N antibodies to SARS-CoV-2 (Elecsys, Roche Diagnostics). Major inclusion criteria were body mass index less than 30 kg m^−2^, age 18–55 years at screening, seronegative for SARS-CoV-2 antibodies and in good general health with no clinically relevant abnormalities (assessed by the investigator) and negative urine pregnancy test at screening visit and birth control use during the study in women of childbearing potential. Major exclusion criteria were as follows: (1) any significant acute or chronic medical or psychiatric condition, including any unexplained, autoimmune or immunosuppressive disorder; (2) administration of any medication known to interfere with immune responses; (3) administration of any vaccine in the period 30 d before the first study vaccination and up to 21 d after the second study vaccination; (4) previous administration of any SARS-COV-2 vaccine at any time before the study; (5) being at high risk of contracting COVID-19; (6) use of any medication with the intention of prophylaxis against SARS-COV-2 infection; (7) history of allergy to any of the constituents of the coronavirus-like particle COVID-19 vaccine, vaccine adjuvants or tobacco; and (8) a history of anaphylactic allergic reactions to plants or plant components.

Healthy seronegative participants 18–55 years of age who met all inclusion criteria and no exclusion criterion (see the protocol in the Supplementary Information for all inclusion/exclusion criteria) were enrolled between 13 July 2020 and 9 August 2020, and were randomized into nine groups in a ratio of 1:1:1:1:1:1:1:1:1 using a permuted block randomization schedule (pre-specified 20 participants per group). Groups 1–3 comprised unadjuvanted CoVLP at three dose levels (3.75 µg, 7.5 µg or 15 µg) or CoVLP at the same dose levels with either AS03 (Groups 4–6) or CpG1018 (Groups 7–9). Given the relatively small group size and the number of experimental questions being addressed (that is, optimal dose, need for an adjuvant and best adjuvant), no formal power calculations were performed. The sample size of ~180 participants with 20 participants in each treatment group was considered to be sufficient to perform an initial evaluation of CoVLP immunogenicity and to detect gross differences in the rates of AEs. The sample size was not large enough to detect all types of AEs, including less frequent or rare events.

For the adjuvanted formulations, CoVLP was mixed with adjuvant immediately before injection, and each particpant received two intramuscular doses in a volume of 0.5 ml 21 d apart (unadjuvanted formulations were administered in a volume of 0.25 ml). The participants and the personnel collecting the safety information and working in testing laboratories remained blinded to treatment allocation. On Day 0 (Day 0: pre-first dose), Day 21 (pre-second dose) and Day 42 (post-second dose), serum and PBMCs were processed for immune outcomes as described previously^29^. All safety information was collected, and all laboratory procedures were carried out, by study staff blinded to treatment allocation. There were no major protocol changes during the conduct of this study before the preparation of the current manuscript.

### Primary and secondary objectives

The primary outcomes focused on the safety/tolerability and immunogenicity of CoVLP administered alone or with one of the two adjuvants used (that is, AS03 and CpG1018) during the 21-d periods after each dose of vaccine. See the protocol in the Supplementary Information for details of all primary, secondary and exploratory outcomes. Primary safety outcomes were the occurrence(s) of: (1) immediate AEs within 30 min after each vaccination; (2) solicited local and systemic AEs up to 7 d after each vaccination; (3) unsolicited AEs, SAEs, AEs leading to withdrawal, AESIs and deaths up to 21 d after each vaccination; (4) participants with normal and abnormal urine and hematological and biochemical values. Primary immunogenicity outcomes were: (1) NAb titers measured using a wild-type MNA and a PNA; and (2) IFN-γ and IL-4 ELISpot responses at 21 d after each dose of vaccine. A secondary safety outcome was the occurrence(s) of SAEs, AEs leading to withdrawal, AESIs and deaths from 22 d after the last vaccination up to the end of the study that is still ongoing. Secondary immunogenicity outcomes were: (1) IgG and/or IgM ELISA titers at 21 d after each dose of vaccine; and (2) all of the immune response outcomes (above) at 201 d and 386 d after the first vaccination. The safety and immunogenicity data collected at later time points in this ongoing study (Day 201 and Day 386) will be released once study follow-up has been completed.

### Safety assessments

For details of safety monitoring, see the protocol in the Supplementary Information. Briefly, enrollment was staggered for dose escalation with sentinel participants at each dose level (*n* = 6) and IDMC review of Day 3 safety data at 10% and 30% recruitment before each dose acceleration. The same process was followed for the second dose. Both passive (diary) and active monitoring of safety signals were performed for the first 42 d of the study and will be continued throughout the study. Active monitoring included telephone contacts with participants 1 and 8 d (Day 1 and Day 8) after each vaccination as well as a site visit on Day 3 after vaccination. Solicited AEs were assessed by the participans as grade 1–4 (mild, moderate, severe or potentially life-threatening) (see Supplementary Table 1 for grading). Unsolicited AEs and AEs leading to participant withdrawal were to be collected up to Day 21 after each vaccination. The following event(s) would pause or halt the study for further review and assessment of the event(s) by the IDMC: (1) any death; (2) any vaccine-related SAE; (3) any life-threatening (grade 4) vaccine-related AE; (4) if 10% or more of participants in a single treatment group experienced the same or similar listed event(s) that could not be clearly attributed to another cause; (5) a severe (grade 3 or higher) vaccine-related AE; (6) a severe (grade 3 or higher) vaccine-related vital sign abnormality; and (7) a severe (grade 3 or higher) vaccine-related clinical laboratory abnormality.

In the event that a pre-defined safety signal was met in any treatment group, at least a transient halt to the study was planned to permit complete evaluation of the reported event(s) and to consult with the IDMC.

All SAEs, AESIs and pregnancies were monitored to Day 42 and will continue to be collected for 6 months after the second dose. Potential cases of VAED, hypersensitivity and pIMD are monitored throughout the study.

### Monitoring for VAED

Any possible safety signals suggestive of VAED after exposure to CoVLP (with or without adjuvants) are being closely monitored throughout the study as follows. AEs within the system organ class list that follows that require inpatient hospitalization (≥24 h) and have laboratory-confirmed SARS-CoV-2 infection are monitored for potential VAED^62,63^. System organ class list: immune system disorders and high-level group term: lower respiratory tract disorders (excluding obstruction and infection), cardiac disorders, signs and symptoms not elsewhere classified (NEC), vascular disorders, heart failures NEC, arteriosclerosis, stenosis, vascular insufficiency and necrosis, cardiac arrhythmias, myocardial disorders and vascular hemorrhagic disorders. High-level term: renal failure and impairment and preferred term: pericarditis, coagulopathy, deep vein thrombosis, pulmonary embolism, cerebrovascular accidents, peripheral ischemia, liver injury, Guillain–Barre syndrome, anosmia, ageusia, encephalitis, chilblains, vasculitis and erythema multiforme (based on standardized Medical Dictionary for Regulatory Activities (MedDRA) classification).

### Monitoring for hypersensitivity reactions

All reported events were also monitored for hypersensitivity reactions after exposure to CoVLP (with or without adjuvants).

In eight clinical studies conducted to date with Quadrivalent VLP Influenza Vaccine (QVLP) candidates produced using similar plant-based technology, all reported events have been monitored for a possible hypersensitivity component (events were searched using both narrow and broad standardized MedDRA queries). Based on these data, there has been a single case of possible early anaphylactic reaction associated with the use of QVLP in humans. A small number of individuals had potential hypersensitivity reactions that were judged to be related to administration of the investigational products (no more than 0.3% of individuals in any given QVLP treatment group experienced such an event), and the events were distributed evenly among treatment groups, including placebo and active comparator groups.

However, because severe reactions are considered to be an important potential risk (based on the theoretical risk that using plants for the production of biotherapeutics might induce hypersensitivity), Medicago required that appropriate medical treatment and supervision were available to manage any possible anaphylactic reactions in this study. To collect data on these events, Medicago closely monitored and assessed allergic reactions that were considered by the site investigators to be related to the investigational product as AESIs.

### Monitoring for pIMDs

Potential immune-mediated diseases are a subset of AEs that include autoimmune diseases and other inflammatory and/or neurologic disorders of interest that might or might not have an autoimmune etiology. The pIMDs that will be monitored specifically are listed in Supplementary Table 2.

### Immunogenicity assessments

#### SARS-CoV-2 spike protein ELISA

Briefly, SARS-Cov2 S protein in its pre-fusion configuration (SARS-Cov2/Wuhan/2019, Immune Technology Corp.: amino acids 1–1,208 with the furin site removed and no transmembrane region) was coated onto a flat-bottom, 96-well microplate at a concentration of 1 µg ml^−1^ in sodium carbonate 50 mM (overnight at 4 °C). After washing steps (PBS-Tween), plates were blocked using Blotto 5% (Rockland) in PBS (1–2 h at 37 °C). After washing steps, serially diluted sera (starting dilution 1/100, fourfold, eight dilutions, in PBS-Tween-Blotto) were added to the wells, in duplicates, and incubated at 37 °C for 1 h. Plates were washed and incubated with secondary antibody (anti-human IgG (H+L) antibody, peroxidase-labeled, SeraCare Life Sciences), diluted at 1/20,000 in PBS-Tween-Blotto and incubated at 37 °C for 1 h. Plates were washed and incubated with peroxidase substrate (SureBlue TMB, SeraCare Life Sciences) for 20 min at room temperature. Reactions were stopped using hydrochloric acid, and absorbance was read at 450 nm within 2 h (Varioskan Flash microplate reader, Thermo Fisher Scientific). Optical density (OD) results for the serial dilutions were used to generate a four-parameter logistic regression. The titer was defined as the reciprocal dilution of the sample for which the OD is equal to a fixed cutpoint at the lower limit of detection. Samples below the cutpoint were attributed a value of 50 (half the minimum required dilution). This is a qualified assay (for example, precision, reproducibility and robustness), but its sensitivity for detecting the full spectrum of COVID-19 disease is not currently known. The specificity of this assay was evaluated using a panel of well-defined sera (that is, COVID--negative and COVID-positive controls as well as samples from individuals infected with other common human coronaviruses). The ELISA results reported in this work are consistent with previous reports of low-level cross-reactivity with other human betacoronaviruses in a wide range of ELISA tests^64^.

#### SARS-CoV-2 PNA

NAb analysis was performed using a cell-based pseudotyped virus neutralization assay (Nexelis). Pseudotyped virus particles were generated using a genetically modified VSV backbone from which the glycoprotein G was removed and luciferase reporter introduced (rVSVΔG-luciferase, Kerafast) to allow quantification using relative luminescence units (RLU). This rVSVΔG-luciferase virus was transduced into HEK293T cells that had previously been transduced with the full-length SARS-CoV-2 S glycoprotein (NXL137-1 in POG2 containing 2019-nCOV Wuhan-Hu-1; GenBank: MN908947) from which the last 19 amino acids of the cytoplasmic tail were removed (rVSVΔG-luciferase-spike ΔCT). Serial dilutions (starting dilution of 1/10, twofold, eight dilutions, in complete growth media) of the heat-inactivated human sera (56 °C for 30 min) were prepared in a 96-well plate in duplicates. The SARS-CoV-2 pseudovirus (in complete growth media) was added to the sera dilutions to reach a target concentration equivalent to approximately 150,000 RLU per well, and the mixture was incubated at 37 °C with 5% CO_2_ supplementation for 1 h. Serum–pseudovirus complexes were then transferred onto plates previously seeded overnight with Vero E6 cells (ATCC CRL-1586), expressing ACE-2 receptor, and incubated at 37 °C with 5% CO_2_ supplementation for 20–24 h. Once incubation was completed, cells were lysed, and samples were equilibrated using the ONE-Glo EX Luciferase Assay System (Promega), incubated for 3 min at room temperature, and the luminescence level was read using a luminescence plate reader (i3× plate reader, Molecular Devices). The resulting RLU was inversely proportional to the level of NAbs present in the serum. For each sample, the neutralizing titer was defined as the reciprocal dilution corresponding to the 50% neutralization (NT_50_) when compared to the pseudoparticle control. The NT_50_ was interpolated from a linear regression using the two dilutions flanking the NT_50_. Samples below the cutoff were attributed a value of 5 (half the minimum required dilution). This is a qualified assay (for example, precision, reproducibility and robustness), but its sensitivity for the full spectrum of COVID-19 disease is not currently known. Its precise specificity is also unknown, although it has been evaluated using a panel of well-defined sera (that is, COVID-negative and COVID-positive controls as well as samples from individuals infected with other common human coronaviruses).

#### SARS-CoV-2 microneutralization cytopathic effect-based assay

NAb analysis was also performed using a cell-based cytopathic effect (CPE) assay as previously described (VisMederi)^65^. Sera samples were first heat inactivated (56 °C for 30 min) and then serially diluted (starting dilution of 1/10, twofold, eight dilutions, in complete growth media). Wild-type SARS-Co-2 virus (2019 nCOV ITALY/INMI1, provided by EVAg; GenBank: MT066156) was then added at a final concentration of 25 TCID_50_ per ml (in complete growth media), and plates were incubated for 1 h at 37 °C with 5% CO_2_ supplementation. At the end of the incubation, the mixture was transferred onto duplicate 96-well microtiter plates pre-seeded overnight with Vero E6 cells (ATCC CRL-1586) expressing ACE-2 receptor to form a uniform monolayer. Plates were then incubated for 3 d at 37 °C with 5% CO_2_ supplementation. After incubation, each well was read under an inverted optical microscope and evaluated for the presence of CPE in at least 50% of the cells contained in the well. In this assay, there is typically an abrupt ‘on–off’ transition between no CPE and destruction of virtually the entire monolayer at one higher dilution. The neutralization titer was defined as the reciprocal of the highest sample dilution that protects at least 50% of the cells from CPE (NT_50_). If no neutralization was observed, samples were attributed a titer value of 5 (half the minimum required dilution). This is a qualified assay (for example, precision, reproducibility and robustness), but its sensitivity is not known for the full spectrum of COVID-19 disease. Its precise specificity is also not known at the current time, although it has been evaluated using a panel of well-defined sera (that is, COVID-negative and COVID-positive controls as well as samples from individuals infected with other common human coronaviruses).

### IFN-γ ELISpot

Cell-mediated immune response was evaluated using an IFN-γ ELISpot assay (human IFN-γ ELISpot assay, Cellular Technology Limited). Cryopreserved PBMCs were rapidly thawed and allowed to rest between 2 and 3 h at 37 °C with 5% CO_2_ supplementation, in CTL-Test media supplemented with 1% glutamine and 1% penicillin–streptomycin. Cells were enumerated and dispensed at 0.5 × 10^6^ cells per well, in duplicates, onto PVDF filter plates pre-coated with an IFN-γ-specific capture antibody. Cells were stimulated using a pool of peptides (15-mer peptides with 11-amino acid overlaps) covering the full sequence of SARS-CoV-2 S protein (USA-CA2/2020, GenBank: MN994468.1, GenScript, purity >90%) at a concentration of 2.19 µg ml^−1^ for 18–24 h at 37 °C with 5% CO_2_ supplementation. After washes (PBS-Tween), biotinylated anti-IFN-γ detection antibody was added to the plates and incubated for 2 h at room temperature, after which, after another round of washes, a streptavidin–alkaline phosphatase conjugate was added and incubated for 30 min at room temperature. After washing steps, substrate solution was added and incubated at room temperature for 15 min, after which the plate was rinsed and left to air dry pending spot enumeration, using an ELISpot reader (ImmunoSpot S6 Universal Analyzer, Cellular Technology Limited). The mean of peptide pool stimulation duplicates was calculated and normalized using the mean of the negative control replicates (control media) and multiplied by a factor of 2 to express cell counts per million cells. Because PBMCs were not available from all participants at Day 0, and because the median value for Day 0 IFN-γ responses across all groups was less than the lower limit of detection of the assay used, the pooled Day 0 results were used to assess vaccine-attributable S protein-specific responses at Day 21 and Day 42.

### IL-4 ELISpot

Cell-mediated immune response was evaluated using an IL-4 ELISpot assay (human IL-4 ELISpot assay, Cellular Technology Limited). Cryopreserved PBMCs were rapidly thawed and allowed to rest between 2 and 3 h at 37 °C with 5% CO_2_ supplementation, in CTL-Test media supplemented with 1% glutamine and 1% penicillin–streptomycin. Cells were enumerated and dispensed at 0.5 × 10^6^ cells per well, in duplicates, onto PVDF filter plates pre-coated with an IL-4-specific capture antibody. Cells were stimulated using a pool of peptides (15-mer peptides) overlapping the full sequence of SARS-CoV-2 S protein (USA-CA2/2020, GenBank: MN994468.1) at a concentration of 2.19 µg ml^−1^ for 32–48 h at 37 °C with 5% CO_2_ supplementation. After washes (PBS-Tween), biotinylated anti-IL-4 detection antibody was added to the plates and incubated for 2 h at room temperature, after which, after another round of washes, a streptavidin–alkaline phosphatase conjugate was added and incubated for 30 min at room temperature. After washing steps, substrate solution was added and incubated at room temperature for 15 min, after which the plate was rinsed and left to air dry pending spot enumeration, using an ELISpot reader (ImmunoSpot S6 Universal Analyzer, Cellular Technology Limited). The mean of peptide pool stimulation duplicates was calculated and normalized using the mean of the negative control replicates (control media) and multiplied by a factor of 2 to express cell counts per million cells. Because PBMCs were not available from all participants at Day 0, and because the median value for Day 0 IL-4 responses across all groups was less than the lower limit of detection of the assay used, the pooled Day 0 results were used to assess vaccine-attributable S protein-specific responses at Day 21 and Day 42.

### Convalescent serum and plasma samples

Sera from COVID-19 convalescent patients were collected from a total of 35 individuals with confirmed disease diagnosis. Time between the onset of the symptoms and sample collection varied between 27 and 105 d. Three samples were collected by Solomon Park, and 20 samples were collected by Sanguine BioSciences; all were from non-hospitalized individuals. Eleven plasma samples were collected from previously hospitalized patients at McGill University Health Centre. Disease severity was ranked as mild (COVID-19 symptoms without shortness of breath), moderate (shortness of breath reported) and severe (hospitalized). These samples were analyzed in parallel of clinical study samples, using the assays described above. Demographic characteristics are provided in Supplementary Table 3.

### Analysis of populations and statistical analysis plan

The full statistical analysis plan can be found in the Supplementary Information. Overall, 180 healthy SARS-CoV-2 seronegative male and female particfipants 18–55 years of age were randomized in a 1:1:1:1:1:1:1:1:1 ratio into nine treatment groups. Randomization was managed by Cytel with Medicago oversight using the Suvoda interactive randomization tool (Suvoda Software v2.2.0). The sample size (20 participants per group) made it possible to perform the initial evaluation of the vaccine immunogenicity and detect major differences in rates of AEs among groups. The sample size was not large enough to detect all types of AEs, including less frequent or rare AEs. The analyses of all immunogenicity endpoints were performed using the per-protocol set. The per-protocol set consists of all randomized participants who received the CoVLP COVID-19 candidate vaccine and completed the study with no major deviations related to participant eligibility, the ability to develop a valid immune response, prohibited medication use or the immunogenicity analyses (Day 21 *n* = 179 and Day 42 *n* = 177). Immunogenicity was evaluated by humoral immune response (NAb assays and IgG ELISA) and cell-mediated immune (CMI) response (ELISpot) induced in participants on Day 0, Day 21 and Day 42. To assess the humoral immune response, the GMT was calculated and compared between adjuvanted and unadjuvanted groups using a crossed two-way analysis of variance (ANOVA) on the log-transformed titers with actual dose level and actual adjuvant type as main effect. The log-transformation was used to meet the normal assumption for the ANOVA. At each time point, the GMT and corresponding 95% confidence interval (CI) of each treatment were obtained by exponential back-transformation of the least square mean. Pairwise comparisons were performed using Tukey’s adjustment method. All possible treatment pairwise comparisons were performed using Tukey’s adjustment method. The geometric mean fold rise (GMFR) at Day 21 or Day 42 was calculated using an analysis of covariance on the difference between Day 21/42 and Day 0 of the log-transformed titer values, with actual dose level and adjuvant type as main effect, dose level by adjuvant type interaction and log-transformed baseline titer as covariate. The GMFR (and corresponding 95% CI) was obtained for each treatment by exponential back-transformation (anti-log with power 10) of the least square means (and corresponding 95% CI). Fisher’s exact test was used to compare the seroconversion rate among the treatment groups. The 95% CI for seroconversion was calculated using the exact Clopper–Pearson method. Pairwise comparisons will be performed without any adjustment method. The specific Th1 and Th2 CMI responses induced on Day 0, Day 21 and Day 42 were measured by the number of T cells expressing IFN-γ and IL-4, respectively, using ELISpot, for each treatment group using a non-parametric method. The overall treatment, dose level and adjuvant type comparisons of the T cells number at Day 21 and Day 42 were compared using the Kruskal–Wallis test, based on asymptotic chi-square distribution of the test statistic. Wilcoxon rank-sum test based on asymptotic normal distribution of the test statistic was used for all pairwise treatment comparisons at a given time point. The comparison between pooled data at Day 0 and Day 21 or Day 0 and Day 42 presented for each treatment group was performed using a Wilcoxon rank-sum test. The relationship between neutralization of pseudovirion and live virus at Day 21 and Day 42 was assessed using a simple linear regression, and the correlation was evaluated using the Pearson correlation coefficient. Safety assessments are based on the Safety Analysis Set—that is, all participants who received either the CoVLP candidate vaccine with or without an adjuvant. Occurrence and incidence of safety events were reported for each treatment group. No formal hypothesis-testing analysis of AE incidence rates was performed, and results were not corrected for multiple comparisons.

### Reporting Summary

Further information on research design is available in the Nature Research Reporting Summary linked to this article.

## Online content

Any methods, additional references, Nature Research reporting summaries, source data, extended data, supplementary information, acknowledgements, peer review information; details of author contributions and competing interests; and statements of data and code availability are available at 10.1038/s41591-021-01370-1.

## Supplementary information

Supplementary Information Supplementary Tables 1–15, Figs. 1 and 2, Clinical Protocol and Statistical Analysis Plan.

Reporting Summary

## Data Availability

Medicago is committed to providing access to anonymized data collected during the trial that underlie the results reported in this article, at the end of the clinical trial, which is currently scheduled to be 1 year after the last participant is enrolled, unless granted an extension. Medicago will collaborate with its partners (GlaxoSmithKline and Dynavax Technologies) on such requests before disclosure. Proposals should be directed to wardb@medicago.com or landryn@medicago.com. To gain access, data requestors will need to sign a data access agreement, and access will be granted for non-commercial research purposes only. The following publicly available databases were accessed to complete this work: GISAID database (https://www.gisaid.org/) and GenBank (https://www.ncbi.nlm.nih.gov/genbank/).
