# Supplementary material for: Phase 1 randomized trial of a plant-derived virus-like particle vaccine for COVID-19
Source: Nat Med. 2021 May 18;27(6):1071–8. doi: 10.1038/s41591-021-01370-1 (PMC8205852; doi:10.1038/s41591-021-01370-1)
Supplement: Supplementary file 2 — Reporting Summary [file 41591_2021_1370_MOESM2_ESM.pdf]

nature research

Corresponding author(s): Brian J Ward

Last updated by author(s): Dec 11, 2020

Reporting Summary

Nature Research wishes to improve the reproducibility of the work that we publish. This form provides structure for consistency and transparency in reporting. For further information on Nature Research policies, see our Editorial Policies and the Editorial Policy Checklist.

Statistics

For all statistical analyses, confirm that the following items are present in the figure legend, table legend, main text, or Methods section.

n/a	Confirmed	
☐	☒	The exact sample size (*n*) for each experimental group/condition, given as a discrete number and unit of measurement
☒	☐	A statement on whether measurements were taken from distinct samples or whether the same sample was measured repeatedly
☐	☒	The statistical test(s) used AND whether they are one- or two-sided<br>*Only common tests should be described solely by name; describe more complex techniques in the Methods section.*
☒	☐	A description of all covariates tested
☐	☒	A description of any assumptions or corrections, such as tests of normality and adjustment for multiple comparisons
☐	☒	A full description of the statistical parameters including central tendency (e.g. means) or other basic estimates (e.g. regression coefficient) AND variation (e.g. standard deviation) or associated estimates of uncertainty (e.g. confidence intervals)
☐	☒	For null hypothesis testing, the test statistic (e.g. *F*, *t*, *r*) with confidence intervals, effect sizes, degrees of freedom and *P* value noted<br>*Give P values as exact values whenever suitable.*
☒	☐	For Bayesian analysis, information on the choice of priors and Markov chain Monte Carlo settings
☒	☐	For hierarchical and complex designs, identification of the appropriate level for tests and full reporting of outcomes
☒	☐	Estimates of effect sizes (e.g. Cohen's *d*, Pearson's *r*), indicating how they were calculated

Our web collection on statistics for biologists contains articles on many of the points above.

Software and code

Policy information about availability of computer code

Data collection: *Provide a description of all commercial, open source and custom code used to collect the data in this study, specifying the version used OR state that no software was used.*

Data analysis: *Provide a description of all commercial, open source and custom code used to analyse the data in this study, specifying the version used OR state that no software was used. (bjw - Graphpad Prism: version 8.1.1)*

For manuscripts utilizing custom algorithms or software that are central to the research but not yet described in published literature, software must be made available to editors and reviewers. We strongly encourage code deposition in a community repository (e.g. GitHub). See the Nature Research guidelines for submitting code & software for further information.

Data

Policy information about availability of data

All manuscripts must include a data availability statement. This statement should provide the following information, where applicable:

- Accession codes, unique identifiers, or web links for publicly available datasets
- A list of figures that have associated raw data
- A description of any restrictions on data availability

bjw - Data availability statement included. The public databases accessed have also been included in this section: GISAID (https://www.gisaid.org) and Genbank (https://www.ncbi.nlm.gov/genbank)

Field-specific reporting

Please select the one below that is the best fit for your research. If you are not sure, read the appropriate sections before making your selection.

☒ Life sciences ☐ Behavioural & social sciences ☐ Ecological, evolutionary & environmental sciences

For a reference copy of the document with all sections, see nature.com/documents/nr-reporting-summary-flat.pdf

Life sciences study design

All studies must disclose on these points even when the disclosure is negative.

Sample size	Group size and overall number of subjects are clearly outlined as well as subject disposition throughout the study
Data exclusions	Subject disposition throughout the study is fully described
Replication	N/A in a clinical trial. All testing was performed using either qualified or validated assays
Randomization	Randomization is clearly described and both the Protocol and Statistical Analysis Plan are provided as Supplemental Material
Blinding	Blinding is clearly described and the Protocol is provided as Supplemental Material

Reporting for specific materials, systems and methods

We require information from authors about some types of materials, experimental systems and methods used in many studies. Here, indicate whether each material, system or method listed is relevant to your study. If you are not sure if a list item applies to your research, read the appropriate section before selecting a response.

Materials & experimental systems

n/a	Involved in the study	
☐	x	Antibodies
☐	x	Eukaryotic cell lines
☒	☐	Palaeontology and archaeology
☒	☐	Animals and other organisms
☐	☒	Human research participants
☐	☒	Clinical data
☒	☐	Dual use research of concern

Methods

n/a	Involved in the study	
☒	☐	ChIP-seq
☒	☐	Flow cytometry
☒	☐	MRI-based neuroimaging

Human research participants

Policy information about studies involving human research participants

Population characteristics	Subject demographics fully described
Recruitment	This information is not specifically mentioned in the manuscript as submitted but can easily be added. It is not stated anywhere that these adult subjects all signed informed consent but this statement can also be added.
Ethics oversight	Although this study was approved by appropriately-constituted research ethics boards (REB) and closely moniteored by an independent data monitoring committee (IDMC) , only the details of IDMC oversight are specifically mentioned in the manuscript as submitted. Details of REB approval/oversight can easily be dded.

Note that full information on the approval of the study protocol must also be provided in the manuscript.

Clinical data

Policy information about clinical studies

All manuscripts should comply with the ICMJE guidelines for publication of clinical research and a completed CONSORT checklist must be included with all submissions.

Clinical trial registration	The trial was registered at clintrials.gov: NCT04450004
Study protocol	The full protocol is provided
Data collection	These details are not provided in the manuscript as submitted but can be added easily

Outcomes

All of the outcomes of the study are fully described in the Protocol (provided) and the primary outcomes are highlighted in the manuscript as submitted
